# Isolated Area Postrema Syndrome Presenting as Intractable Nausea and Vomiting

**DOI:** 10.7759/cureus.7058

**Published:** 2020-02-20

**Authors:** Vasuki Dandu, Suman Siddamreddy, Sreenath Meegada, Vijayadershan Muppidi, Tejo Challa

**Affiliations:** 1 Neurology, Baptist Health Medical Center, Little Rock, USA; 2 Internal Medicine, Baptist Health Medical Center, North Little Rock, USA; 3 Internal Medicine, The University of Texas Health Science Center/Christus Good Shepherd Medical Center, Longview, USA; 4 Internal Medicine, Indiana University Health, Muncie, USA

**Keywords:** nausea, vomiting, neuromyelitis optica, devic's disease, neuromyelitis optica spectrum disorders

## Abstract

Neuromyelitis optica (NMO) is a disease of central nervous system, characterized by demyelination and axonal damage mostly involving optic nerves and spinal cord. Usually these patients present with symptoms related to optic neuritis or myelitis with a typical relapsing course. Some patients present with less common symptoms involving brain stem like nausea and vomiting, especially those involving area postrema (AP) located in dorsal medulla. International panel for NMO diagnosis revised criteria in 2015 and came up with a unifying term NMO spectrum disorders (NMOSD) instead of NMO. Patients with NMO having AP involvement are grouped under area postrema syndrome (APS). Usually patients with AP symptoms also have neurological symptoms upon presentation. Here we present a rare case of an NMO who presented with isolated APS with no other neurological symptoms.

## Introduction

Neuromyelitis optica (NMO) is an inflammatory demyelinating disease of central nervous system involving the optic nerves and spinal cord [1]. The clinical presentation, neuroimages, immunology, and histo-pathological characteristics are distinct from multiple sclerosis [2]. NMO carries poor prognosis than multiple sclerosis [1]. Most of the patients present with symptoms related to optic nerve or spinal cord involvement. Our patient presented only with intractable nausea and vomiting.

## Case presentation

A 39-year-old Asian female presented to primary care physician office with nausea and vomiting for five days. Her symptoms started after she ate a chicken sandwich at a restaurant. She denied any fever, abdominal pain, headache, diarrhea or weakness. No other sick contacts. Her past medical history was only significant for hypothyroidism. Her exam was essentially normal with no abnormal findings.

Labs were significant for mildly elevated blood urea nitrogen (BUN) of 23 and creatinine (Cr) of 1.1. The patient was advised to drink more fluids and was started on proton pump inhibitor.

Patient’s symptoms persisted, so she was referred to a gastroenterologist. Ultrasound abdomen showed normal gallbladder with no stones and a small ruptured ovarian cyst on left. Esophago-duodenoscopy as well as computerized tomography (CT) of abdomen with intravenous and oral contrast showed normal findings.

**Figure 1:**
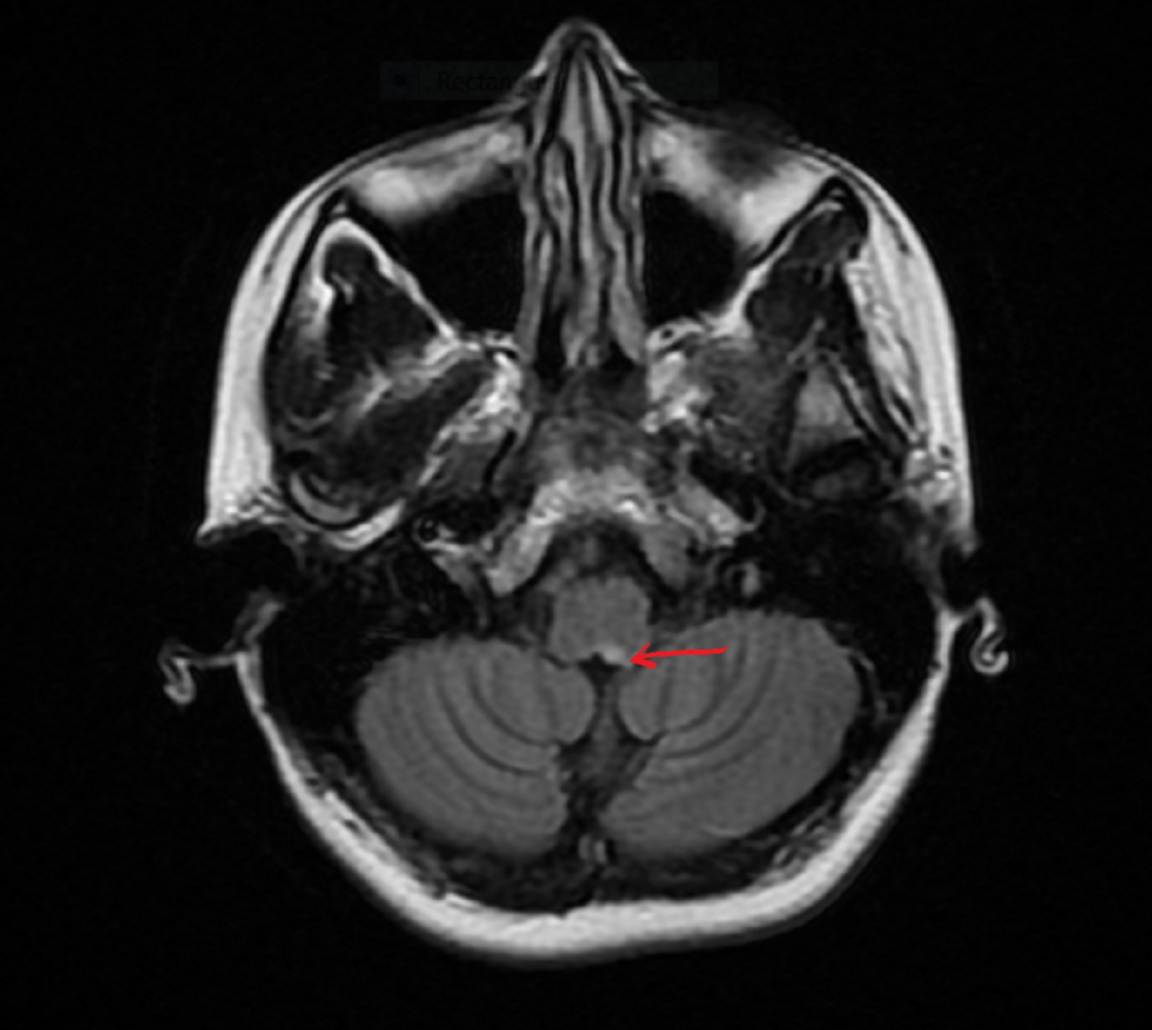
MRI brain FLAIR sequence showing demyelinating lesion in left medulla-area postrema (arrow pointing).

**Figure 2:**
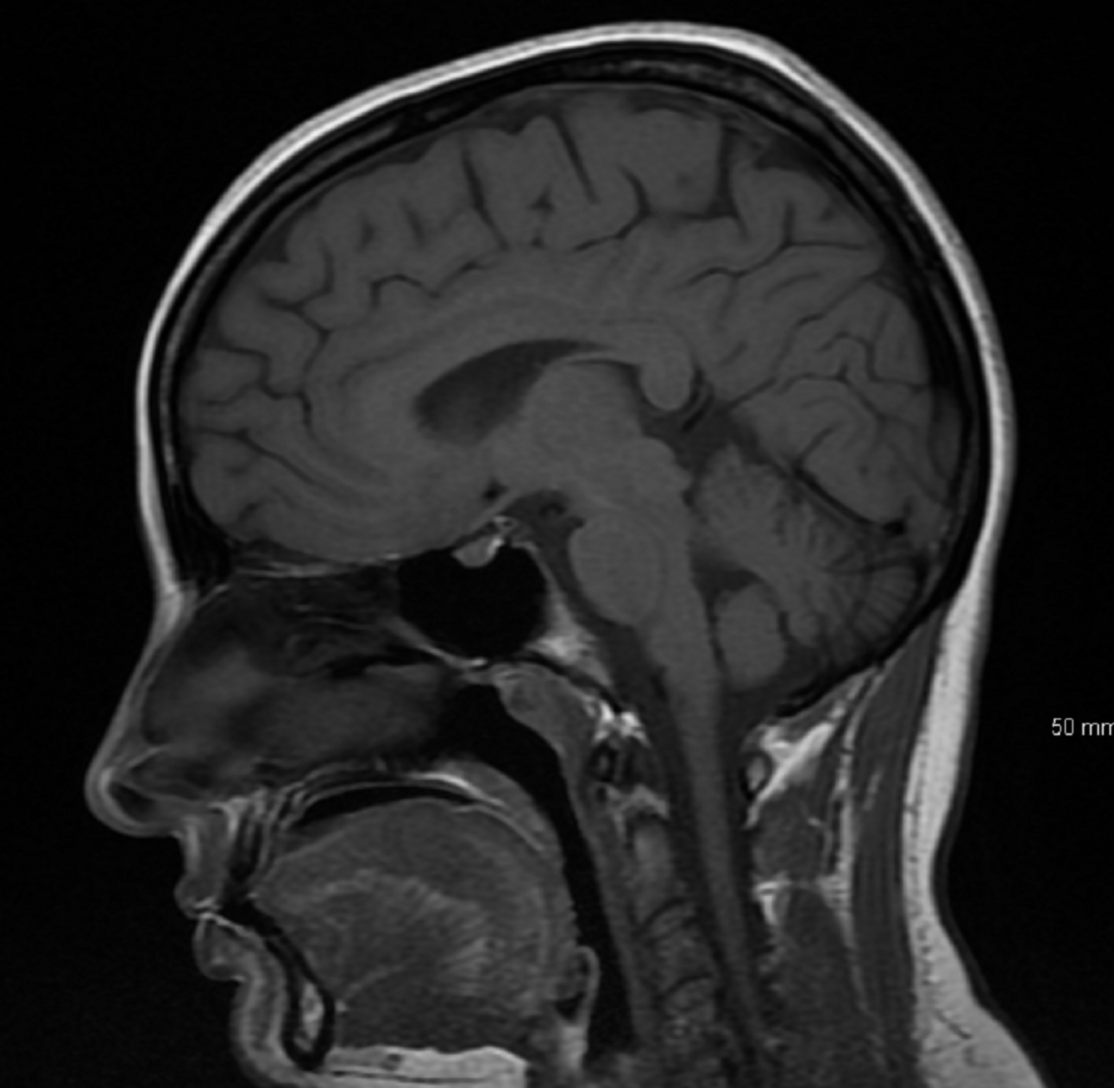
MRI brain sagittal section showing normal brain with no enhancement (Coronal section however showed hyperintense lesion as in Figure 1).

The patient continued to have intractable nausea and vomiting not responding to anti-emetics, so a magnetic resonance imaging (MRI) of brain with and without contrast was done to rule out any central causes. It showed hyperintense lesion in the left dorsal medulla near area postrema (Figures 1, 2).

Neurologist referral was made and she was tested positive for Aquaporin 4 (APQ-4) antibodies in her serum-14.1 U/ml (normal is less than 3 U/ml). She was then diagnosed as having neuromyelitis optica syndrome (NMOSD). Spinal tap was not done as she fit the criteria for NMOSD.

**Figure 3:**
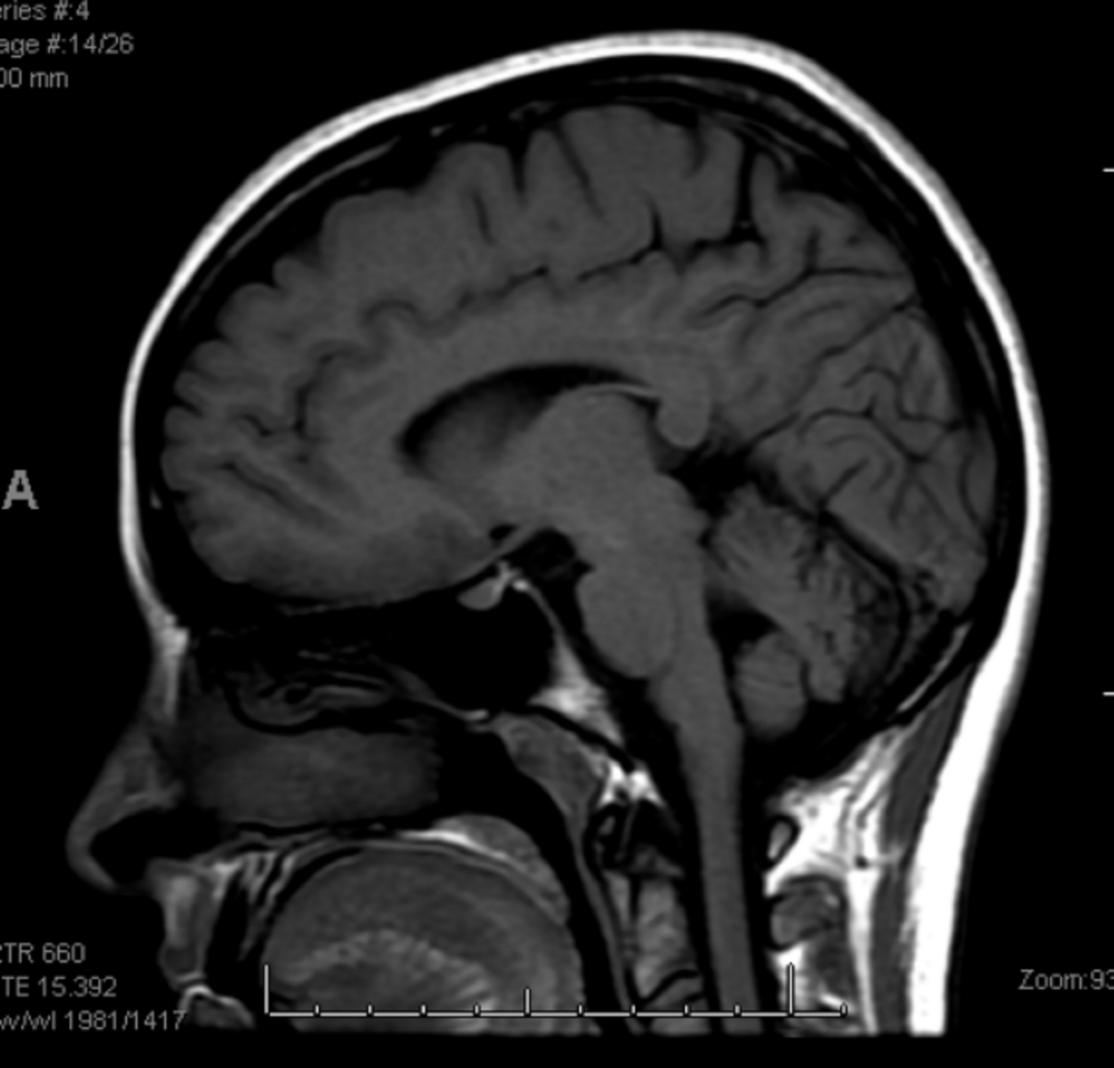
Repeat MRI brain sagittal section did not show any enhancing/hyperintense lesions.

**Figure 4:**
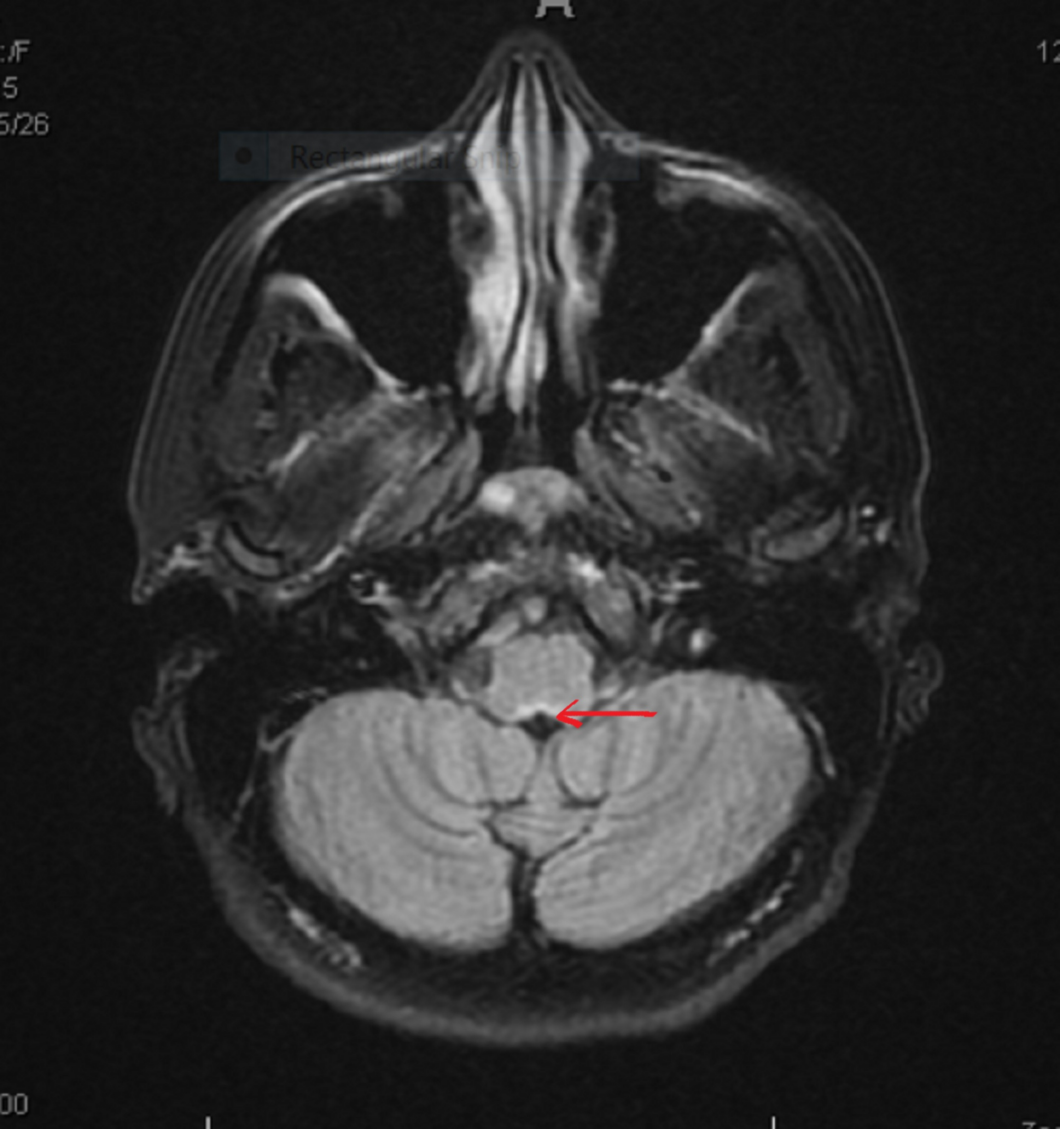
Repeat MRI brain coronal section showed only old lesion at area postrema, no new lesions (arrow pointing).

The patient did not have any neurological symptoms at that time. Arrangements were made for intravenous methylprednisolone infusion at an outpatient infusion center for following week. Meanwhile, the patient developed acute tongue deviation to the right and presented to a local emergency room. She was diagnosed with acute hypoglossal nerve palsy which is a part of NMOSD clinical spectrum. Repeat MRI brain with and without contrast did not show any new lesions (Figures 3, 4).

MRI cervical spine with and without contrast was also done which did not show any demyelinating lesions. She was then emergently treated with IV methyl-prednisone 1000 mg x five days in the hospital and discharged home on oral tapering dose of prednisone. The patient was then evaluated by a multiple sclerosis specialist and was started on oral Methotrexate. Later during an OP follow-up, patient’s nausea and vomiting completely resolved, but still had some residual tongue deviation to right.

## Discussion

NMO prevalence is about 0.5-10 per 100,000 people [1]. It is more common in females with a median age of onset between 32.6-45.7 years [2]. It is a disorder mediated by humoral immune system [3]. Most of the patients will have positive serum NMO-IgG antibody or Aquaphorin-4 autoantibody [4]. Only 1/3 of patients with NMO present with brain stem syndromes [5]. Brain lesions are typical in areas like AP which has high AQP4 expression [6]. Some patients also test positive for Myelin-oligodendrocyte glycoprotein antibody [7].

AP is an area at floor of fourth ventricle which has capillaries with loose endothelial junctions which are rich in AQP4 water channels [8]. Lesions in AP have more inflammation in contrast to spinal and optic lesions which have more demyelination and necrosis. That is probably the reason that patients with AP involvement from NMOSD usually have complete resolution of symptoms with treatment. Detailed history and thorough physical examination, not only focusing on primary symptoms, is essential as there can be concomitant autoimmune disorders in patients with AQP-4 antibody positive NMO [9, 10].

Hallmark symptoms of NMO include visual loss, limb weakness, sensory loss and bladder dysfunction with a remitting-relapsing course. Occasionally some patients present with nausea, vomiting, and hiccups. Symptoms, like the ones described above are characteristic, while none of them are disease specific, so clinical judgement is always necessary. A study from Mayo clinic revealed that 14% of their patients who were diagnosed with NMOSD had nausea and vomiting as their initial presentation. Most of the patients developed other neurological symptoms as the disease progressed.

Basic laboratory tests that are recommended for diagnosis and exclusion of differential diagnoses include: complete blood count, serum chemistry, vitamin B12, folic acid, blood glucose, urine analysis, antibodies associated with auto immune disorders like ANA, anti-ds-DNA, lupus anticoagulant, ANCA, anti-phospholipid antibodies, Treponema pallidum assay, paraneoplastic antibodies, etc. Serum testing for AQP-4 antibodies and myelin oligodendrocyte glycoprotein antibodies is really important. Original AQP-4 assay showed 73% sensitivity and 91% specificity for detection of NMOSD [11].

Diagnosis is primarily based on presence of core clinical characteristics, AQP-4 antibody status, and MRI features. International panel for NMO diagnosis revised criteria in 2015 and came up with the following guidelines for diagnosis as outlined in Table 1 [12].

**Table 1:** NMOSD diagnostic criteria for adults. AQP4: Aquaporin 4; IgG: Immunoglobulin; LETM: Longitudinally extensive transverse myelitis lesions; NMOSD: Neuromyelitis optica spectrum disorders. Modified from [12].

Diagnostic criteria for NMOSD with AQP4-IgG	
1. At least one core clinical characteristic	
2. Positive AQP4-IgG	
3. Exclusion of other/alternative diagnoses	
Diagnostic criteria for NMOSD without AQP4-IgG or NMOSD with unknown AQP4-IgG status	
1. At least two core clinical characteristics occurring as a result of one or more clinical attacks and meeting all the below requirements:	
a) At least one core clinical characteristic must be optic neuritis, acute myelitis with LETM, or area postrema syndrome	
b) Dissemination in space (two or more different core clinical characteristics)	
c) Fulfillment of additional MRI requirements, as applicable	
2. Negative tests for AQP4-IgG using best available detection method or testing unavailable	
3. Exclusion of alternative diagnoses	
Core Clinical Characteristics	
1. Optic neuritis	
2. Acute myelitis	
3. Area postrema syndrome: episode of otherwise unexplained hiccups or nausea and vomiting	
4. Acute brain stem syndrome	
5. Symptomatic narcolepsy or acute diencephalic clinical syndrome with NMOSD-typical diencephalic MRI lesions	
Additional MRI requirements for NMOSD without AQP4-IgG and NMOSD with unknown AQP4-IgG status	
1. Acute optic neuritis requires brain MRI showing (a) normal findings or only nonspecific white matter lesions, OR (b) optic nerve MRI with T2-hyperintense lesion or T1-weighted gadolinium-enhancing lesion extending over >1/2 optic nerve length or involving optic chiasm.	
2. Acute myelitis requires associated intramedullary MRI lesion extending over three contiguous segments (LETM) OR greater than or equal to three contiguous segments of focal spinal cord atrophy in patients with history compatible with acute myelitis.	
3. Area postrema syndrome requires associated dorsal medulla/area postrema lesions.	
4. Acute brain stem syndrome requires associated periependymal brainstem lesions.	

Acute exacerbations should be promptly treated with high-dose methyl prednisone - 1 gm intravenous infusion daily for 3-5 days [13]. For patients unresponsive or partially responsive to steroids, therapeutic plasma exchange is recommended for up to total of seven exchanges [14]. As NMO has a relapsing course, long-term immune suppressive therapy should be initiated after initial treatment. Several immunosuppressive agents have been recommended for treatment to prevent relapses. For attack prevention, long-term immunotherapy is recommended [15]. Azathioprine was one of the first agents that showed efficacy in preventing relapses [16]. Rituximab and Mycophenolate also have been shown to reduce relapses [17].

Recently FDA approved eculizumab to prevent relapses in NMO patients. In a recent study it was shown that patients with NMO and positive AQP-4 antibodies who received eculizumab had a significantly lower risk of relapse compared to the ones who received placebo [18]. Eculizumab is a human monoclonal antibody which is a C5a inhibitor that blocks activation of complement which plays a key role in NMO. There are some ongoing studies with inebilizumab and satralizumab for prevention of relapses in patients with NMO [19,20].

## Conclusions

Neuromyelitis optica spectrum disorders carry a high morbidity and mortality given its highly recurrent nature. Clinical presentation includes hallmark features of optic neuritis, transverse myelitis, and there are patients like ours with atypical presentation that pose a challenge. Early diagnosis and treatment is really important as this disease carries a risk of severe disability and death.
